# Longitudinal TyG–BMI trajectories predict carotid atherosclerosis progression in a Chinese retrospective cohort

**DOI:** 10.3389/fcvm.2025.1672514

**Published:** 2025-11-28

**Authors:** Congling Wang, Ying Xu, Yongpeng Zhang, Ping Peng, Jing Li, Honghua Ye

**Affiliations:** Department of Cardiology, Ningbo Medical Center Lihuili Hospital, Ningbo, China

**Keywords:** carotid atherosclerosis, TyG–BMI, insulin resistance, cardiovascular risk, trajectory analysis, cohort study

## Abstract

**Background:**

Carotid atherosclerosis (CAS) is a major precursor of ischemic stroke, underscoring the importance of early identification of reliable metabolic risk markers. Insulin resistance and metabolic dysfunction are recognized as key contributors to CAS development, and the triglyceride–glucose body mass index (TyG–BMI) has emerged as a promising surrogate biomarker.

**Methods:**

In this retrospective cohort study, we analyzed 2,329 Chinese adults who underwent serial health examinations, with a median follow-up of 1,826 days. Using latent class trajectory modeling, two distinct TyG–BMI trajectories were identified: stable (Class 1) and rising (Class 2).

**Results:**

CAS progression was observed in 46% of participants, with a higher incidence in the rising trajectory group (52.9% vs. 39.9%). Multivariable Cox regression showed that a rising TyG–BMI trajectory was independently associated with an increased risk of CAS progression (adjusted HR = 1.19, 95% CI: 1.04–1.36), particularly among younger adults (≤50 years, HR = 2.04) and women (HR = 1.88). Sensitivity analyses confirmed robustness. Extended prediction models incorporating trajectories demonstrated good calibration, higher discrimination, and greater clinical net benefit in decision curve analysis.

**Conclusion:**

These findings underscore the value of dynamic TyG–BMI monitoring in vascular risk stratification and targeted prevention. To our knowledge, this is the first large-scale study to establish the predictive value of longitudinal TyG–BMI trajectories for CAS progression in a Chinese population.

## Introduction

Carotid atherosclerosis (CAS), characterized by plaque formation and arterial narrowing, is a major contributor to ischemic stroke and accounts for the majority of stroke-related mortality worldwide (1–5). In China, the prevalence of CAS among adults aged 30–79 years has reached 27.2%, representing a significant public health burden and underscoring the urgent need for effective early prevention strategies and precise risk stratification tools (1–5).

Insulin resistance (IR) is a well-established pathophysiological link between metabolic disorders—such as obesity, metabolic syndrome, and diabetes—and the development of atherosclerosis. IR drives oxidative stress, low-grade inflammation, and endothelial dysfunction, collectively accelerating vascular injury and atherosclerotic progression (6). Although direct measurement of IR by the hyperinsulinemic-euglycemic clamp or the Homeostasis Model Assessment of Insulin Resistance (HOMA-IR) is clinically valid, these approaches are often impractical in large-scale or routine clinical settings due to their complexity and cost (7).

The triglyceride-glucose (TyG) index, calculated from fasting triglyceride and glucose levels, has emerged as an accurate, accessible, and cost-effective alternative for assessing IR. The TyG index demonstrates strong concordance with clamp-derived IR and, in some contexts, outperforms HOMA-IR in identifying insulin resistance (8, 9). It is recognized as a reliable alternative marker with diagnostic performance comparable to HOMA-IR but with greater accessibility (10). Notably, systemic inflammation and adiposity are bidirectionally associated with IR. C-reactive protein (CRP), an established inflammatory marker, shows strong correlations with IR in both diabetic and non-diabetic populations (11, 12). Against this background, the TyG–BMI index—incorporating triglyceride and glucose metabolism with body mass index (BMI)—offers a novel composite indicator for evaluating metabolic dysfunction and cardiovascular risk, particularly in populations with obesity (13).

The TyG index has been widely linked to cardiovascular risk, with longitudinal trajectory studies demonstrating its association with CAS progression (9). However, whether a composite index that also integrates BMI (TyG–BMI) provides superior predictive value for CAS progression remains unclear. TyG–BMI has recently been established as a robust marker of insulin resistance, correlating strongly with hypertension, metabolic syndrome, stroke, and cardiovascular risk in diverse populations, including Japanese and American cohorts (14–17). Studies have shown that TyG–BMI offers predictive value for cardiovascular mortality and metabolic outcomes beyond either TyG or BMI alone, and exhibits nonlinear, U-shaped relationships with adverse outcomes in specific subgroups such as patients with diabetes and chronic kidney disease. Carotid ultrasound is a validated, noninvasive imaging tool for CAS assessment, employing both carotid intima-media thickness (CIMT) and plaque detection, and is recognized as a surrogate marker for cardiovascular risk progression (17, 18).

Most prior studies have relied on single time-point measurements of TyG or TyG–BMI, which may not fully reflect the cumulative and dynamic nature of metabolic risk (9, 13). In reality, individuals' metabolic status fluctuates over time, and a trajectory approach may better capture sustained or progressive metabolic dysfunction that contributes to vascular injury. Thus, assessing the longitudinal patterns of TyG–BMI could provide additional insight into early risk stratification and prevention.

Despite these advances, the dynamic relationship between long-term TyG–BMI trajectories and longitudinal CAS progression remains unexplored. This study aims to address this gap by investigating the association between TyG–BMI trajectories and CAS progression over time using trajectory modeling. Specifically, we seek to determine whether distinct metabolic trajectory patterns—stable vs. rising—predict CAS development, and to evaluate the utility of TyG–BMI trajectory analysis for improving atherosclerotic cardiovascular disease (ASCVD) risk stratification. Ultimately, our findings aim to provide new mechanistic insights and practical tools for the early detection and prevention of cardiovascular disease in clinical practice, thereby supporting precision risk stratification and intervention in clinical cardiology.

## Materials and methods

### Study design and participant recruitment

**Figure 1:**
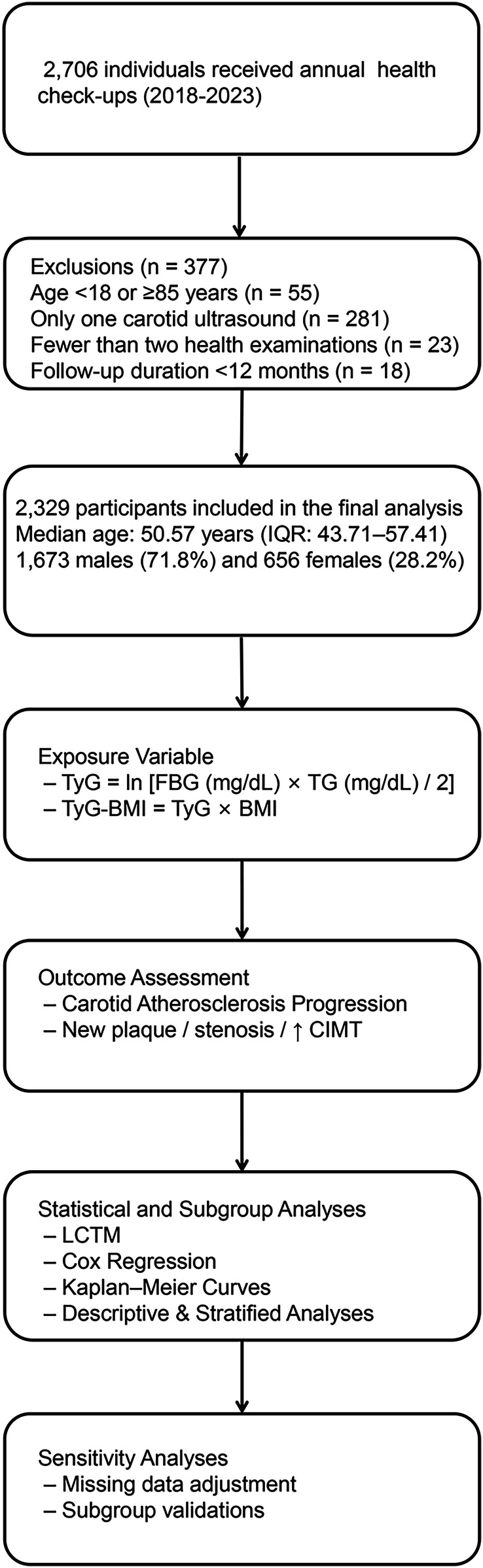
Flowchart of participant selection and analytic framework. Flowchart showing participant selection from 2,706 to 2,329 adults undergoing health examinations (2018–2023), followed by schematic overview of the study design, including exposure definition (TyG–BMI), outcome assessment (carotid atherosclerosis progression), and statistical analyses (trajectory modeling, Cox regression, stratified and sensitivity analyses). Behavioral factors (smoking, alcohol, physical activity) were not recorded due to dataset limitations.

This retrospective cohort study analyzed comprehensive health examination data collected at Ningbo Medical Center Li Huili Hospital between January 2018 and December 2023. Initially, 2,706 adult participants were recruited during routine annual health examinations. To ensure robust and reliable longitudinal analysis, strict inclusion and exclusion criteria were applied (Figure 1).

Participants younger than 18 years or older than 85 years (*n* = 55) were excluded to reduce age-related confounding and improve the generalizability of findings to a stable adult population. Additionally, 281 participants with only one carotid ultrasound measurement, 23 participants with fewer than two physical examinations, and 18 participants with follow-up durations shorter than 12 months were excluded to ensure sufficient temporal resolution and accurate carotid artery assessment. Consequently, 2,329 participants met all inclusion criteria and were included in the final analysis.

The median age of the included cohort was 50.6 years [interquartile range (IQR): 43.7–57.4]. The median follow-up duration was 1,826 days (approximately 60.9 months; IQR: 1,780–1,872 days). Participants underwent annual health examinations according to hospital protocols with high compliance, resulting in minimal loss to follow-up (<5%). Missing data were minimal and did not significantly impact the primary outcomes. To assess the robustness of our findings, sensitivity analyses were conducted by further restricting the cohort to participants with a follow-up duration of at least 24 months, while maintaining the original TyG–BMI trajectory groupings.

The study protocol was reviewed and approved by the Institutional Review Board of Ningbo Medical Center Li Huili Hospital. Due to the retrospective nature of the study, the requirement for written informed consent was waived. The study adhered to the principles of the Declaration of Helsinki and followed the Strengthening the Reporting of Observational Studies in Epidemiology (STROBE) guidelines.

Behavioral and lifestyle factors—including tobacco use, alcohol consumption, dietary habits, and physical activity—were not collected due to dataset limitations, which may introduce residual confounding. This limitation has been addressed in the Discussion.

### Clinical and anthropometric measurements

All data were collected by trained clinical staff following standardized operating procedures to minimize inter-observer variability. A structured and validated questionnaire was administered to record demographic information, including age, sex, and medical history. Anthropometric measurements, including body weight and height, were obtained with participants wearing light clothing and no shoes. Body mass index (BMI) was calculated as weight in kilograms divided by height in meters squared (kg/m^2^).

Blood pressure was measured using an automated oscillometric device after participants had rested in a seated position for at least five minutes. Three consecutive readings were obtained, and the mean value was used for analysis. Hypertension was defined as systolic blood pressure (SBP) ≥ 140 mmHg, diastolic blood pressure (DBP) ≥ 90 mmHg, self-reported physician diagnosis, or current use of antihypertensive medication.

### Laboratory measurements

Fasting venous blood samples (after ≥8 h of fasting) were analyzed at the hospital's central laboratory. Fasting blood glucose (FBG), total cholesterol (TC), triglycerides (TG), HDL-C, LDL-C, white blood cell count (WBC), and ferritin were measured using enzymatic colorimetric assays on a Beckman Coulter automated chemistry analyzer (Beckman Coulter Inc., Brea, CA, USA). Non-high-density lipoprotein cholesterol (non-HDL-C) was calculated as total cholesterol (TC) minus high-density lipoprotein cholesterol (HDL-C).

The TyG–BMI index, which served as the primary exposure variable in this study, was calculated by multiplying the TyG index by body mass index (BMI) (13). The TyG index was calculated as follows (7–9):

TyGindex=ln([FBG(mg/dL)×TG(mg/dL)]/2)

where FBG is fasting blood glucose and TG is triglyceride. The TyG–BMI was then calculated as:

TyG–BMI=TyGindex×BMI

where BMI is body mass index (kg/m^2^).

FBG and TG values measured in mmol/L were converted to mg/dL before calculating the TyG index.

### Assessment of fatty liver

Abdominal ultrasonography was performed by experienced sonographers using a high-resolution Philips EPIQ 7 ultrasound system. Fatty liver diagnosis was based on characteristic sonographic features, including increased liver echogenicity, liver-kidney contrast, and vascular blurring, following established clinical criteria. The presence or absence of fatty liver was recorded and used as a covariate in subsequent analyses.

### Carotid ultrasonography and definition of study outcomes

High-resolution B-mode ultrasonography was conducted bilaterally by a high-frequency (≥7.5 MHz) linear array transducer. Certified sonographers, blinded to participant clinical information and study objectives, performed bilateral carotid artery examinations. Measurements focused on the far wall of the common carotid artery approximately 1 cm proximal to the bifurcation.

Carotid intima-media thickness (CIMT) was measured as the average of three independent readings taken at the far wall of the common carotid artery approximately 1 cm proximal to the bifurcation. According to the Mannheim CIMT Consensus and the American Society of Echocardiography guidelines, a focal thickening of ≥1.5 mm above the adjacent intima-media layer or a CIMT value ≥1.5 mm was classified as a carotid plaque (18). Progression of carotid atherosclerosis (CAS) was defined as the development of new carotid artery stenosis, the emergence of new plaques, or an increase in CIMT during the follow-up period. The overall study design, including exposure assessment, outcome definitions, and analytic strategy, is summarized in Figure 1.

### Covariate assessment

Baseline covariates recorded in 2018 included:

1. demographic variables: age and sex;
2. metabolic indicators: systolic blood pressure (SBP), diastolic blood pressure (DBP), total cholesterol (TC), triglycerides (TG), high-density lipoprotein cholesterol (HDL-C), and low-density lipoprotein cholesterol (LDL-C);
3. glycemic biomarkers: fasting blood glucose (FBG);
4. comorbidities: hypertension and diabetes, defined based on clinical history, diagnostic criteria, or medication use. Diabetes was diagnosed if FBG ≥7.0 mmol/L or if antidiabetic therapy was administered.

All biochemical analyses were conducted in an ISO-accredited laboratory under strict internal and external quality control protocols.

Information on medication use (e.g., statins, antihypertensive, or antidiabetic agents) was not available in the current dataset, as these data were not routinely collected during annual health checkups. This limitation has been acknowledged in the Discussion.

### Statistical analysis

All statistical analyses were performed using R software version 4.5.0. The normality of continuous variables was assessed using the Kolmogorov–Smirnov test. Parametric data are presented as mean ± standard deviation and compared using Student's *t*-test, while non-parametric data are expressed as median (interquartile range) and compared using the Mann–Whitney *U* test for two-group comparisons or the Kruskal–Wallis test for three or more groups. Categorical variables are reported as frequencies (percentages) and compared using the chi-square test. Statistical significance was set at a two-sided *p*-value <0.05.

## Latent class trajectory modeling (LCTM)

Latent Class Trajectory Modeling (LCTM) was conducted using the lcmm package in R to identify distinct longitudinal trajectories of TyG–BMI. This approach classifies individuals into mutually exclusive subgroups based on similarities in temporal TyG–BMI patterns. The optimal number of trajectories was determined by the lowest Bayesian Information Criterion (BIC), posterior probabilities greater than 0.7, and subgroup proportions of at least 5%. Each trajectory was labeled based on its visual and clinical characteristics.

### Cox proportional hazards regression analysis

Participants were stratified into trajectory groups derived from latent class trajectory modeling (LCTM) to explore associations between TyG–BMI dynamics and CAS progression. Kaplan–Meier survival curves were generated for each trajectory group, and group differences were assessed using the log-rank test. Subgroup Kaplan–Meier analyses stratified by age, sex, hypertension, and diabetes status were also performed.

Multivariable Cox proportional hazards regression models were applied to estimate hazard ratios (HRs) and 95% confidence intervals (CIs) for CAS progression. Follow-up time was defined as the interval from baseline examination (time zero) to the first occurrence of CAS progression (event) or the last available follow-up date (censoring). The follow-up duration ranged from 370 to 1,947 days (approximately 12.2 to 63.9 months).

**We first fitted an unadjusted model including trajectory class only.** Adjusted models were then constructed sequentially as follows:

1. **Model 1:** adjusted for age and sex;
2. **Model 2:** additionally adjusted for **non–HDL-C;**
3. **Model 3:** further adjusted for **white blood cell count (WBC)** and **ferritin;**
4. **Model 4:** additionally adjusted for **diabetes;**
5. **Full model:** further adjusted for **hypertension.**

Interaction terms were tested to assess effect modification in subgroup analyses (**age, sex, hypertension, and diabetes**).

### Incremental predictive value, discrimination, calibration, and clinical utility

We compared a baseline Cox model including conventional risk factors with an extended model incorporating the TyG–BMI trajectory class. Individual 60-month risks were obtained from both models. Discrimination and overall performance at 60 months were assessed using time-dependent AUC and the Brier score. The time-dependent AUC assesses the model's ability to distinguish between high- and low-risk individuals over the 60-month period, while the Brier score measures the model's overall prediction accuracy, penalizing large prediction errors. Model calibration at 60 months was evaluated using:

- Bootstrapped calibration plots (using rms::calibrate, B = 300, sample size per resample = 200).
- Calibration slope and calibration-in-the-large (CITL), which assess how well the predicted risks align with actual observed outcomes.

Clinical utility was evaluated using decision curve analysis (DCA) across threshold probabilities of 5% to 50% (step size = 1%) (Supplementary Figure S1).

### Reclassification analyses

At the 60-month horizon (t0 = 60 months), category-free net reclassification improvement (cfNRI) and integrated discrimination improvement (IDI) were calculated to assess the model's ability to improve the reclassification of individuals into more accurate risk categories. Cases were defined as participants who experienced an event by 60 months, and controls were participants with follow-up beyond 60 months. Individuals censored before 60 months were excluded from the analysis to avoid bias in risk classification. Two-sided 95% confidence intervals and *p*-values were obtained via stratified bootstrap (B = 1,000) to assess the robustness of the results. The resulting cfNRI and IDI estimates demonstrated the added value of the trajectory model in risk stratification, highlighting its ability to provide better differentiation of high-risk individuals for CAS progression (Supplementary Table S1).

### Sensitivity and missing data analysis

A series of sensitivity analyses were performed to assess the robustness of the main findings. These analyses included:

1. restricting the cohort to participants with a follow-up duration greater than 24 months, while maintaining the original TyG–BMI trajectory groupings (trajectory classification was not re-estimated in this subset);
2. imputation of missing values for numerical variables with less than 10% missing data, using stratified median imputation based on disease status (e.g., diabetic vs. non-diabetic);
3. exclusion of variables with 10% or more missing data from the analysis;
4. exclusion of participants missing more than one TyG–BMI measurement, while retaining those with two or more measurements; and
5. acknowledgment of potential residual confounding due to unmeasured behavioral factors such as physical activity and smoking. Given the retrospective design, potential information and selection biases cannot be excluded.

Future prospective studies with comprehensive data collection are warranted to validate these findings.

## Results

### Baseline characteristics according to CAS progression

A total of 2,329 participants were included in this longitudinal cohort study. Of these, 1,258 (54.0%) did not show carotid atherosclerosis (CAS) progression during follow-up, while 1,071 (46.0%) exhibited progression. The median age was 50.6 years (IQR: 43.7–57.4), and 71.8% (*n* = 1,673) were male. The median follow-up duration was 1,826 days (approximately 60.9 months; IQR: 1,780–1,872).

Baseline characteristics stratified by CAS progression are summarized in Table 1. Participants with progression were significantly older (54.59 vs. 47.34 years, *p* < 0.001), but the sex distribution was similar between groups (male: 72.5% vs. 71.2%, *p* = 0.488). The progression group had higher systolic and diastolic blood pressure (SBP and DBP), BMI, triglycerides (TG), total cholesterol (TC), low-density lipoprotein cholesterol (LDL-C), and non-high-density lipoprotein cholesterol (non-HDL-C) (all *p* < 0.01). High-density lipoprotein cholesterol (HDL-C) did not differ significantly (*p* = 0.396). Inflammatory markers, including white blood cell counts (*p* = 0.004) and ferritin levels (*p* < 0.001), were elevated in the progression group. Hypertension (16.3% vs. 6.4%) and diabetes (9.0% vs. 1.7%) were more prevalent among those with CAS progression (both *p* < 0.001).

**Table 1:** Baseline demographic, clinical, and laboratory characteristics of participants grouped by carotid atherosclerosis (CAS) progression status.

Variables	Non-progression (*n* = 1,258)	Progression (*n* = 1,071)	*P*-value
Demographic Characteristics			
Age (years)	47.34 [31.02, 52.78]	54.59 [49.87, 63.17]	<0.001
Male, *n* (%)	896 (71.2%)	777 (72.5%)	0.488
Female, *n* (%)	362 (28.8%)	294 (27.5%)	–
Laboratory Indicators			
Triglycerides (mmol/L)	1.37 [1.00, 1.91]	1.52 [1.07, 2.28]	<0.001
Total cholesterol (mmol/L)	5.14 [4.52, 5.73]	5.40 [4.76, 6.12]	<0.001
LDL-C (mmol/L)	2.45 [2.11, 2.84]	2.52 [2.17, 2.91]	0.003
HDL-C (mmol/L)	1.30 [1.11, 1.50]	1.28 [1.09, 1.52]	0.396
non-HDL-C (mmol/L)	3.83 [3.22, 4.39]	4.07 [3.48, 4.76]	<0.001
FBG (mmol/L)	4.97 [4.66, 5.35]	5.38 [4.91, 5.96]	<0.001
TyG index	8.61 [8.28, 8.95]	8.82 [8.44, 9.25]	<0.001
TyG–BMI	197.41 [174.29, 219.28]	209.44 [186.54, 233.11]	<0.001
Inflammatory Markers			
White blood cells (×10^9^/L)	5.90 [5.00, 6.90]	6.10 [5.20, 7.10]	0.004
Ferritin (ng/mL)	130.55 [66.20, 202.20]	142.30 [84.00, 226.15]	<0.001
Comorbidities, *n* (%)			
Hypertension	80 (6.4%)	175 (16.3%)	<0.001
Diabetes	21 (1.7%)	96 (9.0%)	<0.001
Anthropometric parameters			
BMI (kg/m^2^)	22.83 [20.81, 24.89]	23.48 [21.67, 25.40]	<0.001
Physical Examination			
SBP (mmHg)	123.00 [113.00, 134.00]	130.00 [120.00, 142.00]	<0.001
DBP (mmHg)	78.00 [70.00, 85.00]	80.00 [74.00, 88.00]	<0.001

Significant differences were observed between the progression and non-progression groups in metabolic, inflammatory, and comorbidity parameters.

### TyG–BMI trajectory grouping

**Figure 2:**
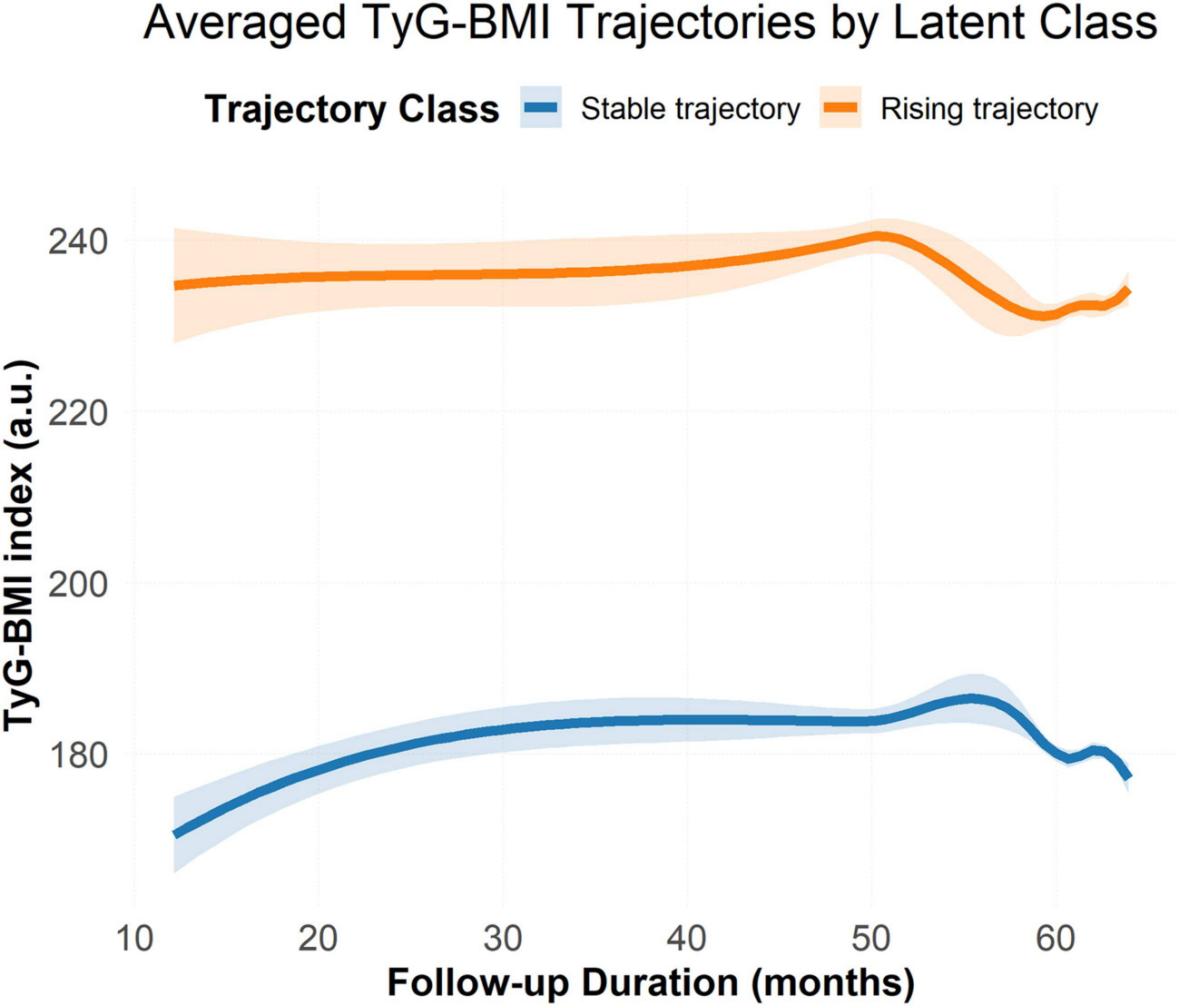
Trajectory analysis of TyG–BMI. Results of latent class trajectory modeling (LCTM) for TyG–BMI over time. Two distinct trajectories were identified: Class 1 (stable, blue) and Class 2 (rising, red). Shaded areas indicate 95% confidence intervals. Trajectory groups were used for subsequent risk analyses.

Latent class trajectory modeling identified two distinct TyG–BMI trajectories (Figure 2; Table 2): Stable Trajectory (Class 1, *n* = 1,238) and Rising Trajectory (Class 2, *n* = 1,091). Class 1 showed relatively stable TyG–BMI levels, whereas Class 2 exhibited a significant upward trend indicating progressive metabolic load.

**Table 2:** Model fit indicators and distribution of TyG–BMI trajectory groups.

Number of groups	Log-likelihood	Parameters	BIC	Group distribution (%)
1	−54,801.45	7	109,657.2	Class 1: 100.0
2	−54,767.43	12	109,627.9	Class 1: 53.16; Class 2: 46.84
3	−54,754.93	17	109,641.7	Class 1: 19.06; Class 2: 13.99; Class 3: 66.94
4	−54,748.27	22	109,667.1	Class 1: 2.62; Class 2: 47.45; Class 3: 2.75; Class 4: 47.19
5	−54,744.75	27	109,698.8	Class 1: 36.80; Class 2: 7.90; Class 3: 40.10; Class 4: 2.62; Class 5: 12.58

This table displays the model fitting results for the latent class trajectory modeling of **TyG–BMI**, including log-likelihood, number of parameters, Bayesian Information Criterion (BIC), and the proportion of participants assigned to each trajectory group.

**Figure 3:**
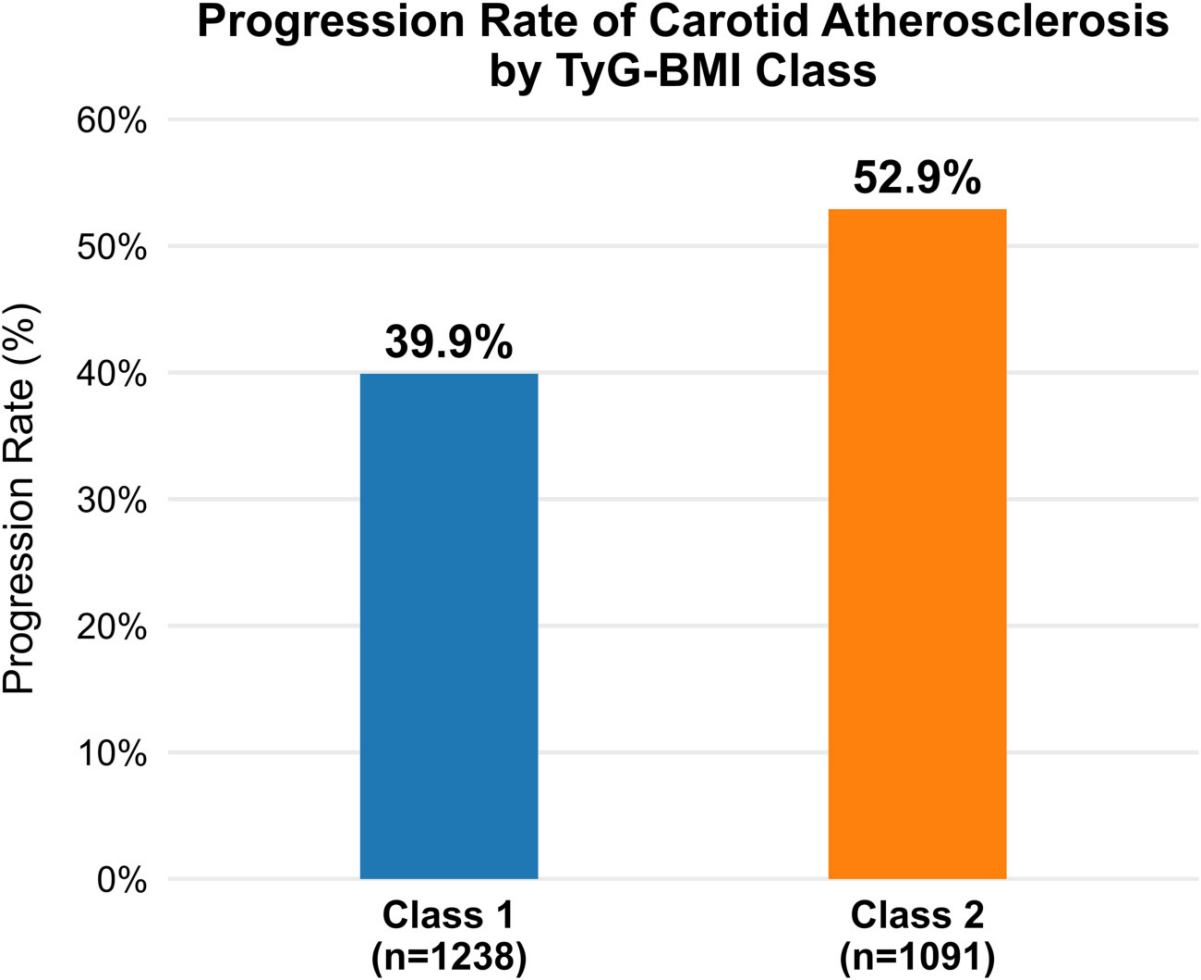
Cumulative density of CAS progression by TyG–BMI trajectory group. Bar graph showing the proportion of participants with carotid atherosclerosis (CAS) progression by TyG–BMI trajectory. Class 2 (rising TyG–BMI) had a higher cumulative incidence of CAS progression than Class 1 (stable TyG–BMI), indicating greater subclinical vascular risk.

Figure 3 illustrates the higher cumulative incidence of CAS progression in Class 2 (rising trajectory) compared with Class 1 (stable trajectory) (52.9% vs. 39.9%, *p* < 0.001).

### Baseline characteristics of TyG–BMI trajectory groups

Baseline features by trajectory group are summarized in Table 3. Age did not differ between classes (*p* = 0.315), whereas **Class 2 (rising)** included a higher proportion of men (*p* < 0.001). Compared with **Class 1 (stable)**, Class 2 showed a less favorable metabolic profile with higher FBG, triglycerides, total cholesterol, LDL-C, non-HDL-C, TyG, and TyG–BMI and lower HDL-C (all *p* < 0.001 except total cholesterol, *p* = 0.001). Inflammatory markers (WBC count and ferritin) were also higher in Class 2 (both *p* < 0.001). Hypertension and diabetes were more common in Class 2 than in Class 1 (both *p* < 0.001). Consistent with these differences, **CAS progression** occurred more frequently in Class 2 (*p* < 0.001).

**Table 3:** Baseline characteristics by TyG–BMI trajectory groups.

Variables	Class 1 (*n* = 1,238)	Class 2 (*n* = 1,091)	*P*-value
Demographics			
Age (years)	50.55 [44.93, 58.79]	50.59 [45.88, 59.67]	0.315
Male, *n* (%)	737 (59.5)	936 (85.8)	<0.001
Female, *n* (%)	501 (40.5)	155 (14.2)	<0.001
Metabolic Parameters			
FBG (mmol/L)	5.04 [4.68, 5.47]	5.25 [4.81, 5.78]	<0.001
Triglycerides (mmol/L)	1.17 [0.88, 1.56]	1.90 [1.38, 2.62]	<0.001
Total cholesterol (mmol/L)	5.20 [4.59, 5.84]	5.33 [4.67, 6.04]	0.001
LDL-C (mmol/L)	2.42 [2.10, 2.79]	2.57 [2.19, 2.97]	<0.001
HDL-C (mmol/L)	1.40 [1.20, 1.62]	1.17 [1.02, 1.35]	<0.001
non-HDL-C (mmol/L)	3.78 [3.20, 4.37]	4.11 [3.54, 4.78]	<0.001
TyG index	8.47 [8.17, 8.77]	9.01 [8.67, 9.35]	<0.001
TyG–BMI	180.75 [166.47, 195.38]	227.77 [213.46, 246.83]	<0.001
Inflammatory Markers			
WBC count (×10^9^/L)	5.60 [4.90, 6.60]	6.40 [5.40, 7.40]	<0.001
Ferritin (ng/mL)	107.40 [52.12, 166.88]	171.60 [108.75, 258.60]	<0.001
Comorbidities, *n* (%)			
Hypertension	88 (7.1)	167 (15.3)	<0.001
Diabetes	42 (3.4)	75 (6.9)	<0.001
CAS progression	494 (39.9)	577 (52.9)	<0.001

This table presents baseline demographic, metabolic, inflammatory, and clinical characteristics of participants stratified by TyG–BMI trajectory class. Class 2 exhibited a significantly higher metabolic burden and CAS progression rate.

### Survival analysis and multivariable Cox regression

**Figure 4:**
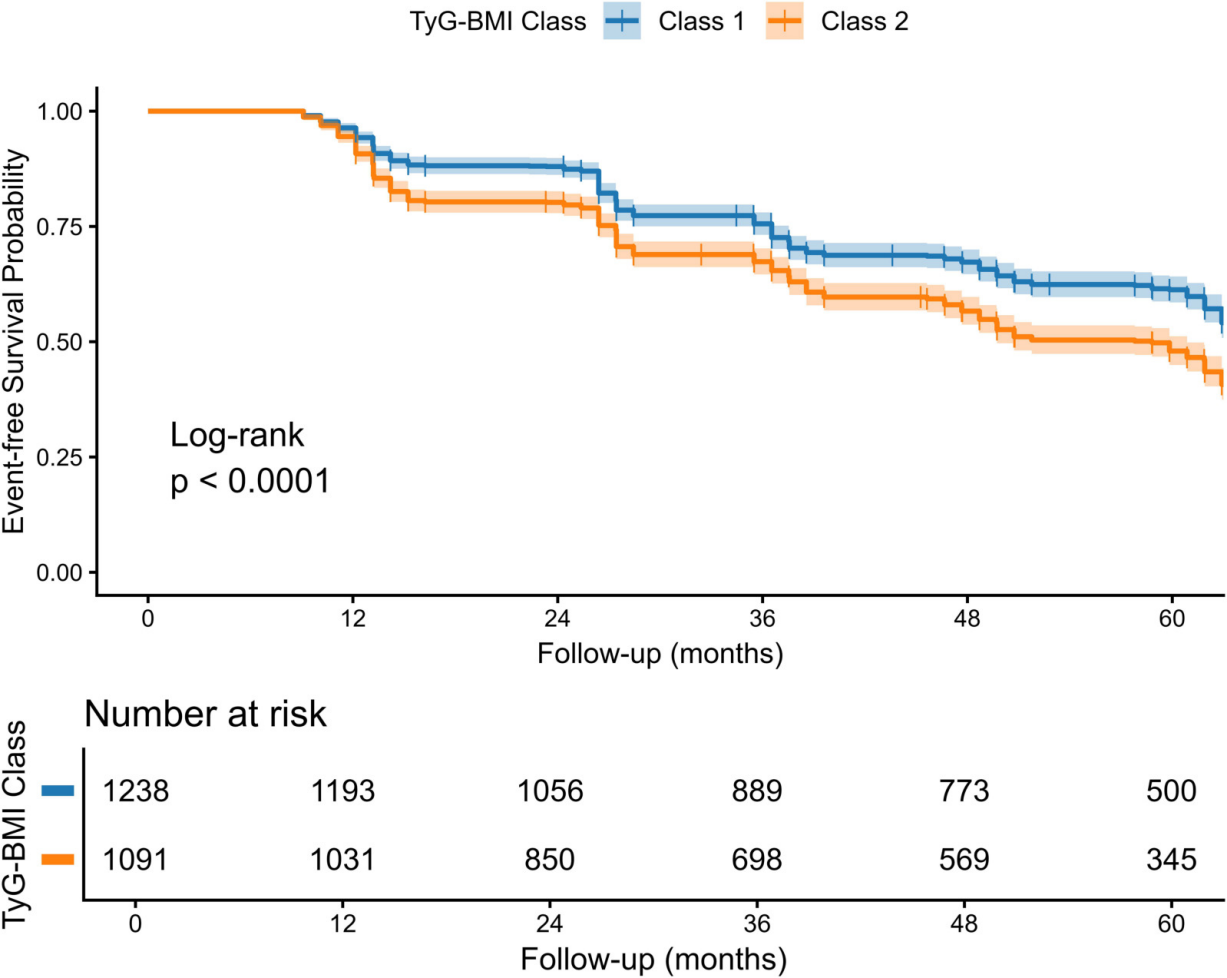
Kaplan–Meier survival curves by TyG–BMI trajectory. Kaplan–Meier curves of **event-free survival (no CAS progression)** by trajectory class over 60 months. Participants in the **rising** TyG–BMI trajectory (Class 2) had lower event-free survival than those in the **stable** trajectory (Class 1) (**log-rank *p*** **<** **0.0001**), indicating a higher risk of CAS progression in Class 2. Shaded bands denote 95% CIs; numbers at risk are shown below.

**Figure 5:**
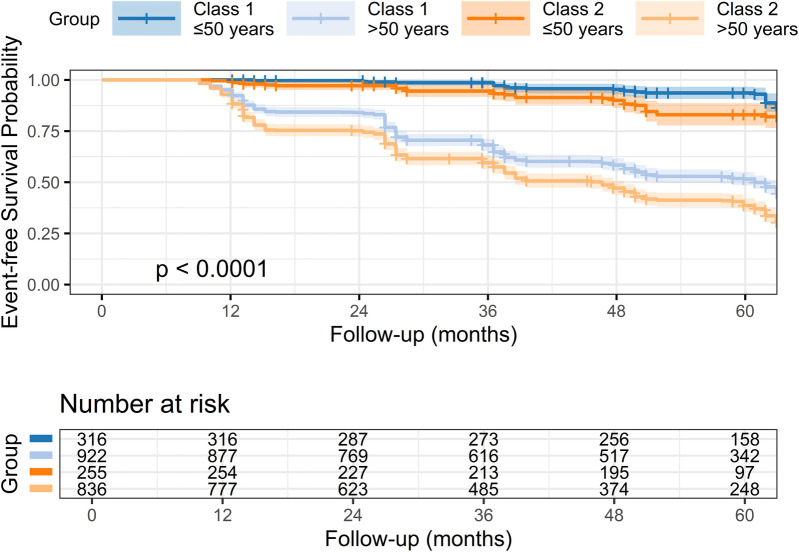
Stratified Kaplan–Meier survival curves by trajectory and age. Survival curves stratified by TyG–BMI trajectory and age (≤50 vs. >50 years). Significant differences in event-free survival were observed among the four groups (log-rank *p* < 0.0001), indicating age-related heterogeneity in the association.

Kaplan–Meier survival analysis demonstrated a significantly lower event-free survival probability in Class 2 compared to Class 1 (log-rank *p* < 0.0001; Figure 4). After stratifying by age, the four curves separated clearly (Figure 5): the poorest survival occurred in **Class 2, >50 years**, whereas the best survival was observed in **Class 1, ≤50 years**.

**Figure 6:**
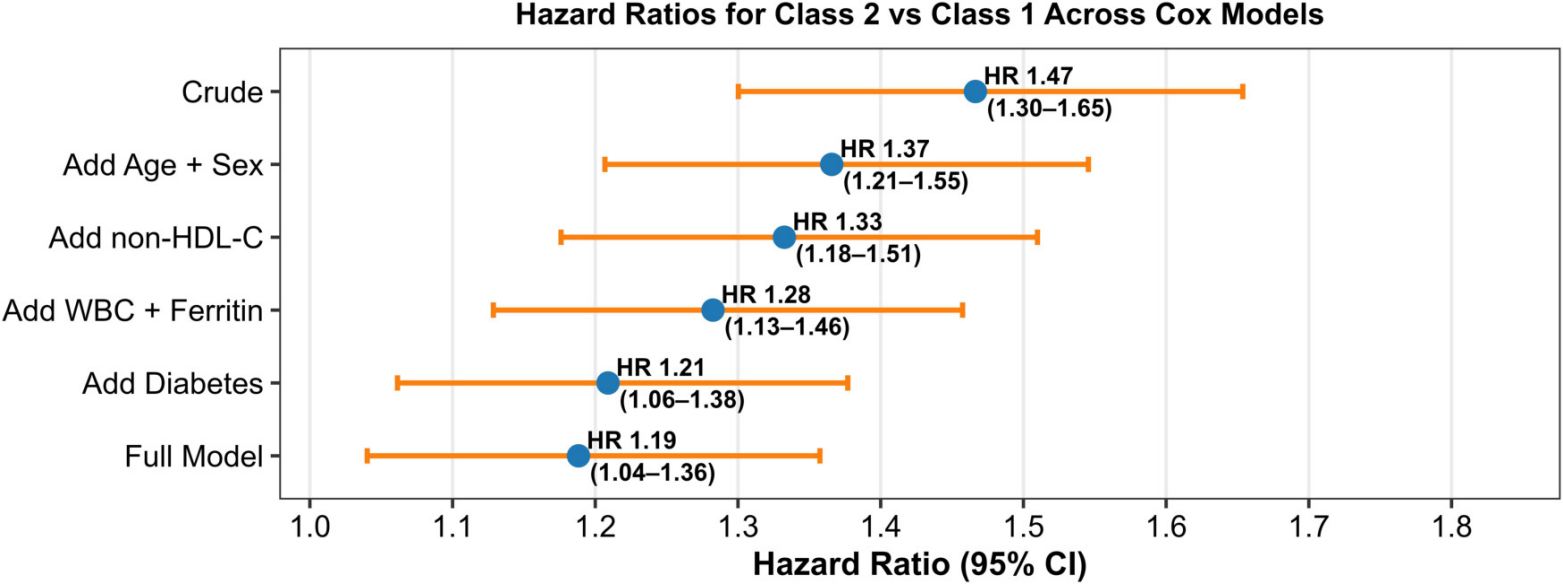
Forest plot of hazard ratios for CAS progression by TyG–BMI trajectory. Forest plot displaying hazard ratios (HRs) and 95% confidence intervals from Cox regression models for the association between rising TyG–BMI trajectory (Class 2) and CAS progression. Covariates were added sequentially; HRs remained statistically significant across all models.

Multivariable Cox proportional hazards models were used to quantify the association between TyG–BMI trajectories and CAS progression (Table 4; Figure 6). In the unadjusted model, participants in Class 2 had a significantly higher risk of CAS progression compared to Class 1 (HR = **1.47, 95% CI: 1.30–1.65; *p*** **<** **0.001**). This risk estimate progressively attenuated but remained statistically significant after stepwise adjustment for potential confounders:

1. HR = **1.37 (95% CI: 1.21–1.55; *p*** **<** **0.001)** after adjustment for age and sex
2. HR = **1.33 (95% CI: 1.18–1.51; *p*** **<** **0.001)** after adding non-HDL-C
3. HR = **1.28 (95% CI: 1.13–1.46; *p*** **=** **0.004)** after additional adjustment for WBC and ferritin
4. HR = 1.21 (95% CI: 1.06–1.38; *p* = 0.011) after adjustment for diabetes
5. HR = **1.19 (95% CI: 1.04–1.36; *p*** **=** **0.011)** in the fully adjusted model including hypertension

**Table 4:** Multivariable Cox proportional hazards models for TyG–BMI trajectory and CAS progression.

Model	Hazard ratio (HR)	95% confidence interval (CI)	*p*-value
Basic Model (Class only)	1.47	1.30–1.65	<0.001
+Age, Sex	1.37	1.21–1.55	<0.001
+Age, Sex + non-HDL-C	1.33	1.18–1.51	<0.001
+WBC + Ferritin	1.28	1.13–1.46	0.004
+Diabetes	1.21	1.06–1.38	0.011
Full Model	1.19	1.04–1.36	0.011

This table displays hazard ratios (HRs) from Cox proportional hazards models evaluating the association between TyG–BMI trajectory class and CAS progression. Adjustment was done sequentially for demographic, metabolic, inflammatory, and comorbidity variables. The association remained significant in most models, indicating the robustness of TyG–BMI as a predictor of vascular risk.

The association between a rising TyG–BMI trajectory and CAS progression remained robust and statistically significant across all models. Notably, the HR decreased from **1.47 in the crude model** to **1.19 in the fully adjusted model**, suggesting partial confounding by metabolic and inflammatory factors. The adjusted hazard ratios across sequential models are illustrated in Figure 6, confirming the consistency and robustness of this association.

### Subgroup analyses

**Figure 7:**
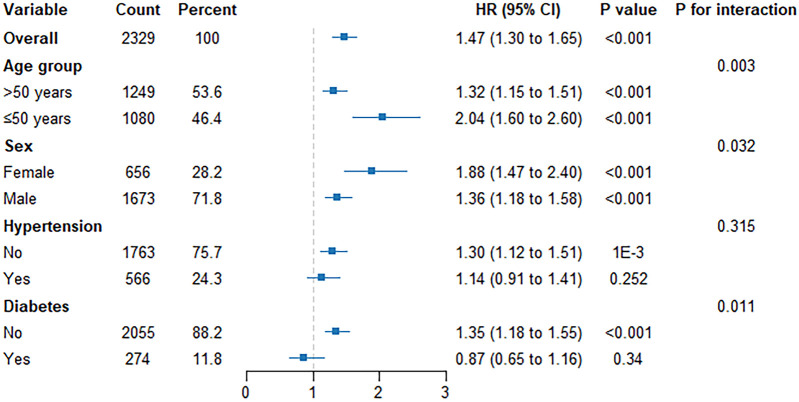
Stratified risk of CAS progression by TyG–BMI and clinical subgroups. Forest plot of adjusted hazard ratios (HRs) comparing the **rising** TyG–BMI trajectory (Class 2) with the **stable** trajectory (Class 1) across age, sex, hypertension, and diabetes subgroups. Risk was higher in participants ≤50 years (HR = 2.04, *p* < 0.001) and in women (HR = 1.88, *p* < 0.001). The association was present in non-hypertensive individuals (HR = 1.30, *p* = 0.001) but not in those with hypertension (HR = 1.14, *p* = 0.252). It was evident in non-diabetic participants (HR = 1.35, *p* < 0.001) but absent in diabetics (HR = 0.87, *p* = 0.34), with significant interactions by age (*p* = 0.003), sex (0.032), and diabetes (0.011), but not hypertension (0.315).

Subgroup analyses (Figure 7) demonstrated that the association between TyG–BMI trajectories and CAS progression varied by age, sex, and diabetes status, but not by hypertension.

### Age stratification:

The risk associated with Class 2 was strongest among participants ≤50 years (HR = 2.04; 95% CI: 1.60–2.60; *p* < 0.001) and remained significant in those >50 years (HR = 1.32; 95% CI: 1.15–1.51; *p* < 0.001), with a significant interaction (*P* for interaction = 0.003).

### Gender stratification

The HR was higher in women (HR = 1.88; 95% CI: 1.47–2.40; *p* < 0.001) than in men (HR = 1.36; 95% CI: 1.18–1.58; *p* < 0.001), with a significant interaction (*P* for interaction = 0.032), suggesting potential sex-specific differences.

### Hypertension stratification

The association was present in non-hypertensive individuals (HR = 1.30; 95% CI: 1.12–1.51; *p* = 0.001) but was not significant in those with hypertension (HR = 1.14; 95% CI: 0.91–1.41; *p* = 0.252); the interaction was not significant (*P* for interaction = 0.315).

### Diabetes stratification

The association was evident in non-diabetic participants (HR = 1.35; 95% CI: 1.18–1.55; *p* < 0.001) but absent in individuals with diabetes (HR = 0.87; 95% CI: 0.65–1.16; *p* = 0.34), with a significant interaction (*P* for interaction = 0.011), suggesting a possible ceiling effect of metabolic injury in diabetes.

As shown in Table 5, female participants in Class 2 (rising TyG–BMI trajectory) had significantly higher rates of hypertension, diabetes, fatty liver, and CAS progression compared with those in Class 1. Moreover, Class 2 females were older and exhibited higher BMI, systolic and diastolic blood pressure, triglycerides, LDL-C, non-HDL-C, TyG, TyG–BMI, white blood cell counts, and ferritin, together with lower HDL-C (most at *p* < 0.01; diabetes remained significant at *p* < 0.05). These findings indicate that female participants with a rising TyG–BMI trajectory experience greater metabolic and inflammatory burden, which may contribute to their increased risk of carotid atherosclerosis progression.

**Table 5:** Baseline and clinical characteristics of female participants stratified by TyG–BMI trajectory group.

Variable	Class 1 female (*n* = 501)	Class 2 female (*n* = 155)	*P*-value
Hypertension (%)	26 (5.2%)	25 (16.1%)	<0.001
Diabetes (%)	21 (4.2%)	15 (9.7%)	0.016
CAS progression (%)	198 (39.5%)	96 (61.9%)	<0.001
Fatty liver (%)	71 (14.2%)	96 (61.9%)	<0.001
BMI (kg/m^2^)	20.87 [19.44, 22.31]	25.00 [23.88, 26.95]	<0.001
SBP (mmHg)	119.00 [110.00, 130.00]	131.03 [121.00, 144.50]	<0.001
DBP (mmHg)	75.00 [69.00, 80.00]	80.00 [72.00, 85.50]	<0.001
WBC (×10^9^/L)	5.40 [4.60, 6.30]	5.80 [5.10, 6.70]	<0.001
TG (mmol/L)	1.07 [0.82, 1.45]	1.66 [1.21, 2.15]	<0.001
LDL-C (mmol/L)	2.44 [2.11, 2.80]	2.62 [2.13, 3.06]	0.008
HDL-C (mmol/L)	1.55 [1.37, 1.74]	1.38 [1.18, 1.54]	<0.001
non-HDL-C (mmol/L)	3.88 [3.33, 4.52]	4.28 [3.54, 5.02]	<0.001
TyG index	8.40 [8.11, 8.69]	8.89 [8.54, 9.27]	<0.001
TyG–BMI	174.94 [161.29, 189.71]	222.45 [212.23, 242.95]	<0.001
Age (years)	50.66 [45.75, 57.48]	56.30 [49.36, 64.65]	<0.001
Ferritin (ng/mL)	54.60 [25.10, 98.30]	83.60 [42.40, 136.05]	<0.001

Data are presented as *n* (%) for categorical variables and median [IQR] for continuous variables. Comparisons were performed using Fisher's exact test (categorical) and Wilcoxon rank-sum test (continuous).

This table presents the demographic, metabolic, inflammatory, and clinical characteristics of female participants, grouped according to TyG–BMI trajectory class (Class 1: stable; Class 2: rising). Data are shown as median [interquartile range, IQR] for continuous variables and *n* (%) for categorical variables. Between-group differences were assessed using the Wilcoxon rank-sum test for continuous variables and Fisher's exact test for categorical variables. Statistically significant differences indicate that women in the rising TyG–BMI trajectory group (Class 2) exhibited higher metabolic burden, more comorbidities, and greater risk of carotid atherosclerosis progression.

Calibration plots and decision curve analyses confirmed that the extended model was well-calibrated and offered greater clinical utility (Supplementary Figure S1).

### Sensitivity analyses

Sensitivity analyses were conducted by restricting the sample to participants with a follow-up duration of at least 24 months, while maintaining the original TyG–BMI trajectory groupings. As shown in Supplementary Figure S2, the event-free survival probability remained significantly lower in Class 2 (rising TyG–BMI trajectory) compared to Class 1 (stable trajectory), consistent with the main findings (log-rank *p* < 0.0001).

In sequential Cox regression models (Supplementary Figure S3), the association between Class 2 and increased risk of CAS progression persisted after adjustment for potential confounders. The results were consistent with the primary analysis, confirming robustness when restricting to participants with at least 24 months of follow-up.

These results further confirm that the association between rising TyG–BMI trajectories and CAS progression is robust and not materially influenced by the exclusion of participants with shorter follow-up durations.

## Discussion

This study demonstrates that longitudinal TyG–BMI trajectories, rather than single baseline values, independently predict the progression of carotid atherosclerosis (CAS). The association was strongest in younger adults and in women, highlighting population-specific vulnerability that may be overlooked in cross-sectional analyses.

Carotid plaques are key contributors to ischemic stroke, and their early detection is central to cardiovascular prevention (19, 20). Insulin resistance (IR) is a well-established driver of atherosclerosis through mechanisms involving endothelial dysfunction, oxidative stress, and inflammation (21, 22). However, direct assessment of IR, such as the euglycemic–hyperinsulinemic clamp or HOMA-IR, is costly and impractical in large populations. The triglyceride–glucose (TyG) index has therefore emerged as a validated, low-cost surrogate of IR with broad clinical applicability, showing predictive value for arterial stiffness and cardiovascular outcomes (7, 9, 10, 23–26).

Prior studies linked the TyG index with carotid atherosclerosis and carotid intima–media thickness (CIMT) progression in both community and clinical cohorts (9, 25). Building on this evidence, our study incorporated obesity into the IR proxy to create TyG–BMI trajectories, which captured cumulative metabolic stress more accurately. The trajectory-based model showed better calibration and higher net clinical benefit in decision curve analysis, supporting its added value for CAS risk stratification.

Our trajectory analysis revealed stronger associations in younger individuals and in women, consistent with previous findings from a Tianjin cohort (10). The relationship remained significant among metabolically “silent” participants without hypertension or diabetes, suggesting that TyG–BMI trajectories may capture early IR-related vascular injury before overt metabolic disease develops.

Our findings complement prior work linking metabolic dysregulation to vascular pathology (27–29). While some studies reported null associations between TyG and carotid disease (28), these discrepancies likely reflect differences in sample size, population age, and analytical approach. The dynamic TyG–BMI trajectories identified here appear to better capture cumulative metabolic stress and thereby provide clearer discrimination of CAS risk.

Supporting this, Wu Z et al. (26) reported a significant association between the TyG index and arterial stiffness progression among hypertensive populations. Notably, Li W et al. (29) examined participants aged over 40 years (mean 60.0 ± 10.8 years), whereas accumulating evidence indicates that subclinical atherosclerosis may begin much earlier, particularly in young and middle-aged adults (30).

Beyond lipid abnormalities, recent studies have further confirmed the predictive value of the TyG index for carotid atherosclerotic burden even among individuals without hyperlipidemia (31). Consistent with these observations, our findings showed that TyG–BMI trajectories predicted CAS progression independent of hypertension or dyslipidemia status. The association was strongest in metabolically “silent” individuals without traditional risk factors, suggesting that dynamic TyG–BMI changes may better capture early insulin-resistance–related vascular injury. In contrast, in hypertensive or dyslipidemic participants, concurrent processes such as endothelial dysfunction, oxidative stress, and vascular inflammation (10, 32, 33) may dilute the relative contribution of TyG-related indices.

### Predictive role of TyG–BMI trajectories in carotid atherosclerosis

Longitudinal changes in TyG–BMI—a composite index integrating obesity (BMI) and insulin resistance (TyG)—were independently associated with carotid atherosclerosis (CAS) progression in this Asian cohort. These findings are in line with previous longitudinal studies showing that TyG-related indices are associated with carotid atherosclerotic burden and arterial stiffness (34–37). Individuals in the rising-trajectory group had a higher risk of CAS progression than those in the stable group, even after adjustment for age, inflammation, and metabolic comorbidities. These findings highlight the independent predictive value of sustained TyG–BMI elevation as a marker of cumulative metabolic stress and support its application as an easily obtainable metabolic indicator in clinical practice (10, 36). Subgroup analyses revealed stronger associations in younger participants and in women, with significant age and sex interactions (Figure 7). The association was weaker in hypertensive participants and absent in those with diabetes, possibly reflecting a saturation or “ceiling” effect of metabolic injury.

### Age- and sex-specific vulnerability

Our findings extend previous reports by showing that TyG–BMI trajectories are particularly informative in younger adults and women. Female participants with rising trajectories exhibited greater metabolic and inflammatory burden, higher ferritin levels, and higher prevalence of fatty liver and CAS progression (Table 5). These results suggest that both metabolic overload and loss of estrogen-mediated vascular protection may contribute to women's increased vascular susceptibility. Early recognition of these high-risk subgroups may offer an important opportunity for prevention and timely intervention.

### Mechanistic insights

The combined increase of BMI and TyG may promote vascular injury through overlapping pathways of oxidative stress and inflammation. Experimental evidence shows that obesity triggers vascular inflammation via lipid-induced activation of TLR4/NF-*κ*B signaling, whereas insulin resistance enhances mitochondrial ROS production (10, 32, 33). Consistent with these mechanisms, individuals in the rising-trajectory group had higher ferritin and WBC levels, supporting the concept that chronic co-exposure to obesity and insulin resistance accelerates atherosclerosis. These findings align with the “metabolic–vascular interaction” and “metabolic memory” hypotheses, in which sustained metabolic stress induces long-term vascular dysfunction via oxidative and inflammatory pathways (10, 38–41).

### Sex differences and hormonal mechanisms

Estrogen exerts vascular-protective effects partly by suppressing pro-inflammatory pathways such as NF-*κ*B, and its decline may increase endothelial vulnerability (42–44). In our study, women with rising TyG–BMI trajectories had significantly higher ferritin levels, suggesting that altered iron metabolism may aggravate vascular injury in postmenopausal females. Experimental evidence further indicates that iron overload can promote degradation of estrogen receptor *α* (ER*α*) through the Mdm2-mediated pathway, thereby weakening estrogen's anti-inflammatory activity and forming a vicious cycle of metabolic and inflammatory injury (45).

### Integration with previous longitudinal evidence

Previous longitudinal studies, such as those by Li et al. (29) and Wu et al. (26), demonstrated that TyG trajectories predict arterial stiffness and carotid atherosclerotic burden. Our results extend this evidence by incorporating BMI to capture total metabolic load. TyG–BMI trajectories predicted CAS progression independent of hypertension or dyslipidemia status and remained significant in metabolically “silent” individuals without conventional risk factors. This suggests that dynamic TyG–BMI changes can detect early vascular injury before overt disease develops (31–33). In contrast, in hypertensive or dyslipidemic participants, additional mechanisms such as endothelial dysfunction, oxidative stress, and vascular inflammation (10, 32, 33) may lessen the relative contribution of insulin-resistance–related indices.

### Predictive and clinical implications

Previous studies have shown that TyG-based indices may improve cardiovascular risk prediction beyond traditional risk scores and inflammatory markers such as high-sensitivity C-reactive protein (hsCRP) (46). In our study, rising TyG–BMI trajectories were predictive of CAS progression even after adjustment for conventional cardiovascular risk factors and inflammation-related markers, and the extended model demonstrated good calibration and higher net clinical benefit in decision curve analysis (Supplementary Figure S1). In line with global cardiovascular prevention strategies and WHO recommendations for accessible, scalable screening tools, serial TyG–BMI assessment represents a practical method for early detection of subclinical vascular risk, particularly among young adults and postmenopausal women (47–51).

## Conclusions

A rising TyG–BMI trajectory is an independent predictor of carotid atherosclerosis progression. The association is particularly strong in younger adults and in women. Dynamic monitoring of TyG–BMI provides incremental value beyond traditional risk factors and may serve as a practical tool for early prevention and precision risk stratification. Future prospective studies integrating behavioral factors, hormonal profiling, and multi-omics analyses are needed to validate these findings and clarify the biological mechanisms underlying trajectory-related vascular injury.

## Limitations

This study has several limitations. First, although ferritin and WBC reflect inflammation and iron status, their tissue-specific effects require validation through experimental studies. Second, hormone levels were not directly assessed, which limits mechanistic interpretation of sex-specific findings. Third, the proposed epigenetic pathways related to metabolic memory were not evaluated and warrant confirmation using molecular or multi-omics approaches. Finally, behavioral factors (smoking, diet, physical activity) and medication use were unavailable in the dataset, which may introduce residual confounding.

## Data Availability

Access to the data can be considered upon reasonable request to the corresponding author and with appropriate institutional approvals. Requests to access these datasets should be directed to Honghua Ye, lhlyehonghua@nbu.edu.cn.

## Supplementary material

Supplementary Figure S1 Model performance at 60 months: calibration and decision curve analysis. (A) Calibration plot comparing predicted versus observed 60-month risks for the baseline Cox model (blue) and the extended model including TyG–BMI trajectory class (orange). The extended model demonstrated closer agreement with observed outcomes, with calibration slope nearer to 1. (B) Decision curve analysis (DCA) showing greater net clinical benefit for the extended model than for the baseline model across threshold probabilities of 5%–50%.

Supplementary Figure S2 Kaplan-Meier curves of event-free survival restricted to participants with at least 24 months of follow-up. Individuals in the rising TyG–BMI trajectory group showed significantly lower event-free survival compared with those in the stable trajectory group (log-rank p < 0.0001).

Supplementary Figure S3 Forest plot of hazard ratios (HRs) for CAS progression in the rising TyG-BMI trajectory group compared with the stable trajectory group, restricted to participants with at least 24 months of follow-up. Stepwise adjustments were performed for demographic, metabolic, inflammatory, and comorbidity factors. The results remained consistent with the primary analysis, supporting the robustness of the association.

Supplementary Table S1 Reclassification analyses at 60 months (cfNRI and IDI).
