# Supplementary figures and images for: Longitudinal TyG–BMI trajectories predict carotid atherosclerosis progression in a Chinese retrospective cohort
Source: Front Cardiovasc Med. 2025 Nov 28;12:1672514. doi: 10.3389/fcvm.2025.1672514 (PMC12698576; doi:10.3389/fcvm.2025.1672514)

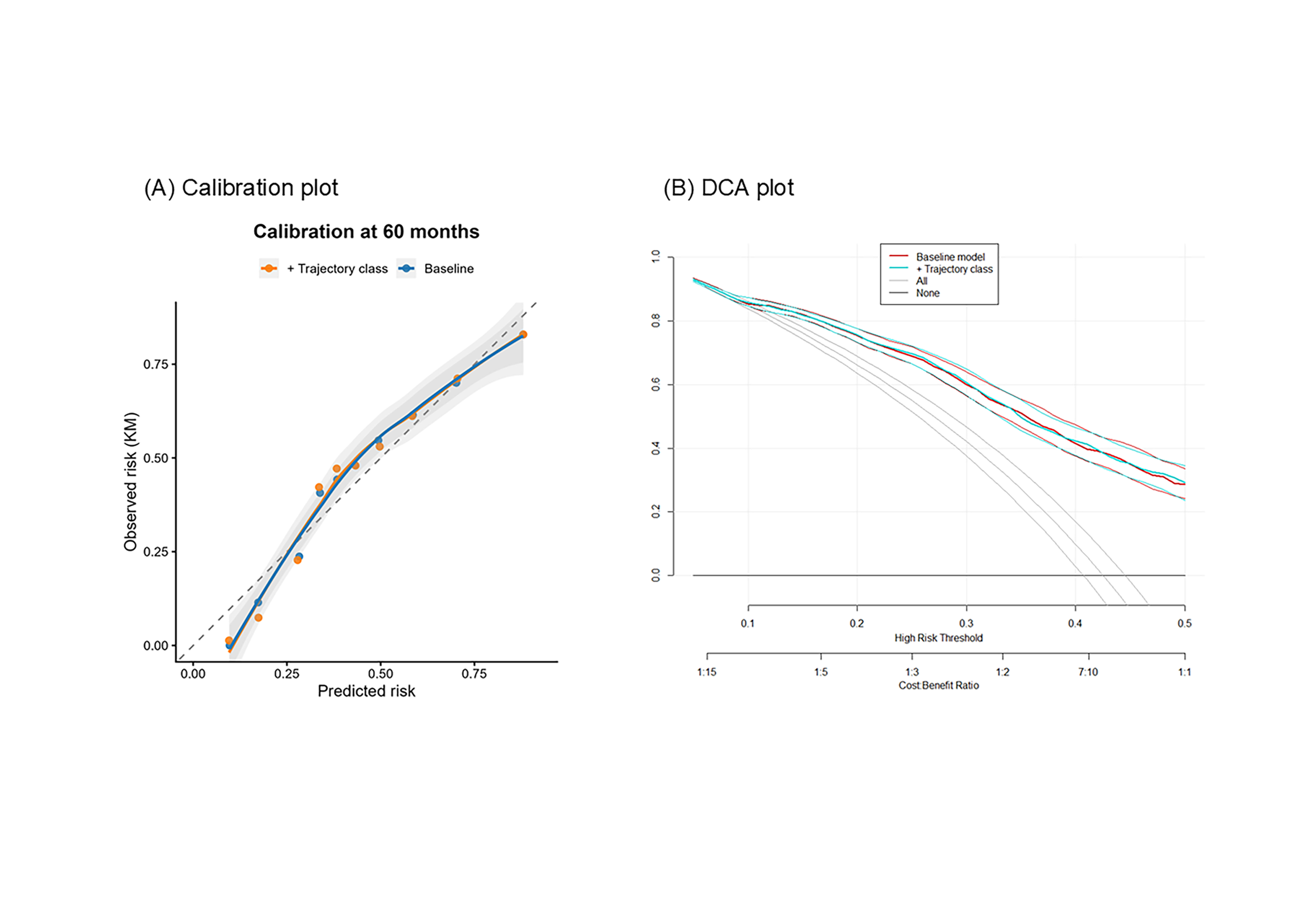

Supplement: Supplementary Figure S1 — Model performance at 60 months: calibration and decision curve analysis. (A) Calibration plot comparing predicted versus observed 60-month risks for the baseline Cox model (blue) and the extended model including TyG–BMI trajectory class (orange). The extended model demonstrated closer agreement with observed outcomes, with calibration slope nearer to 1. (B) Decision curve analysis (DCA) showing greater net clinical benefit for the extended model than for the baseline model across threshold probabilities of 5%–50%. [file Image1.tif]

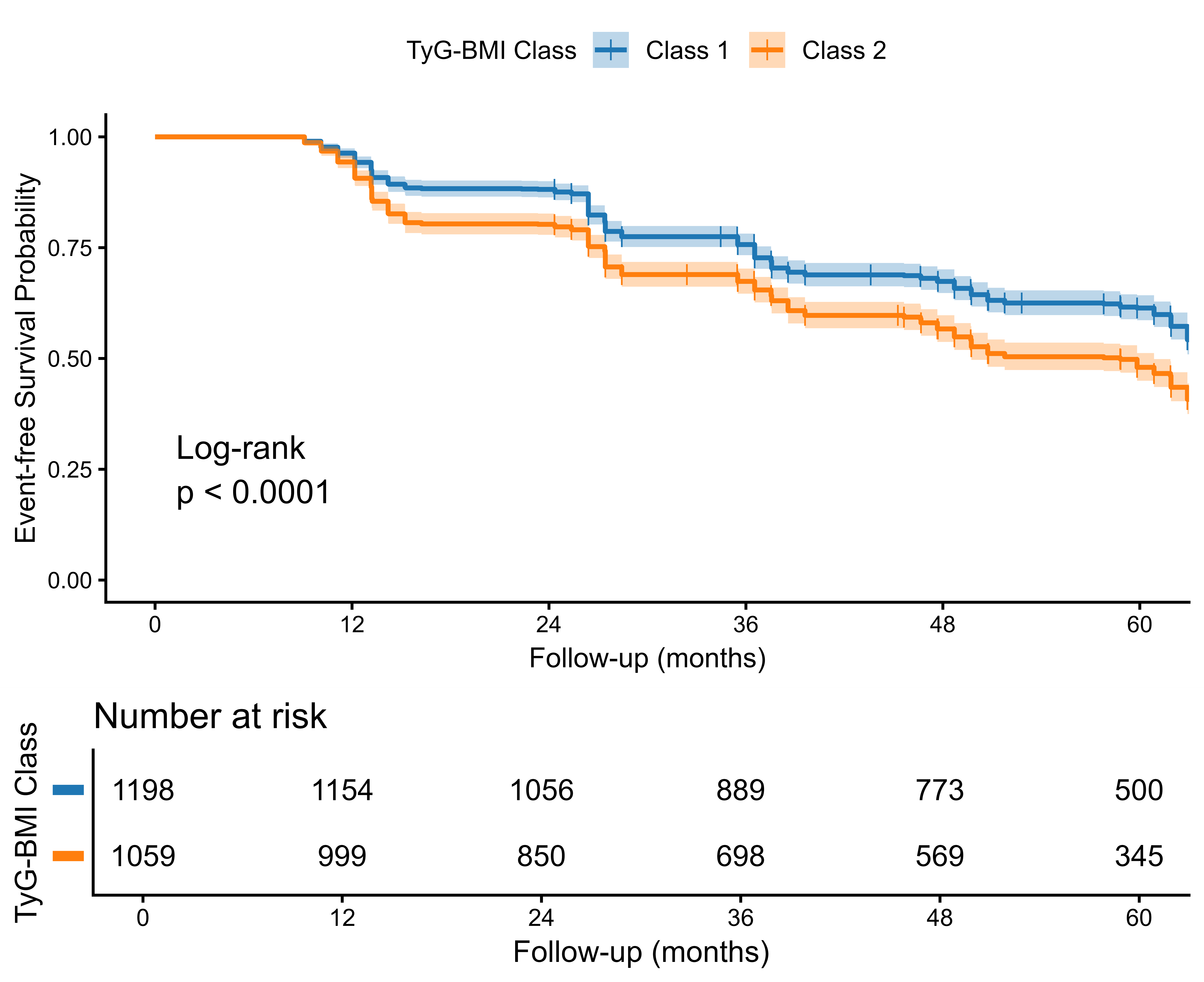

Supplement: Supplementary Figure S2 — Kaplan-Meier curves of event-free survival restricted to participants with at least 24 months of follow-up. Individuals in the rising TyG–BMI trajectory group showed significantly lower event-free survival compared with those in the stable trajectory group (log-rank p < 0.0001). [file Image2.tiff]

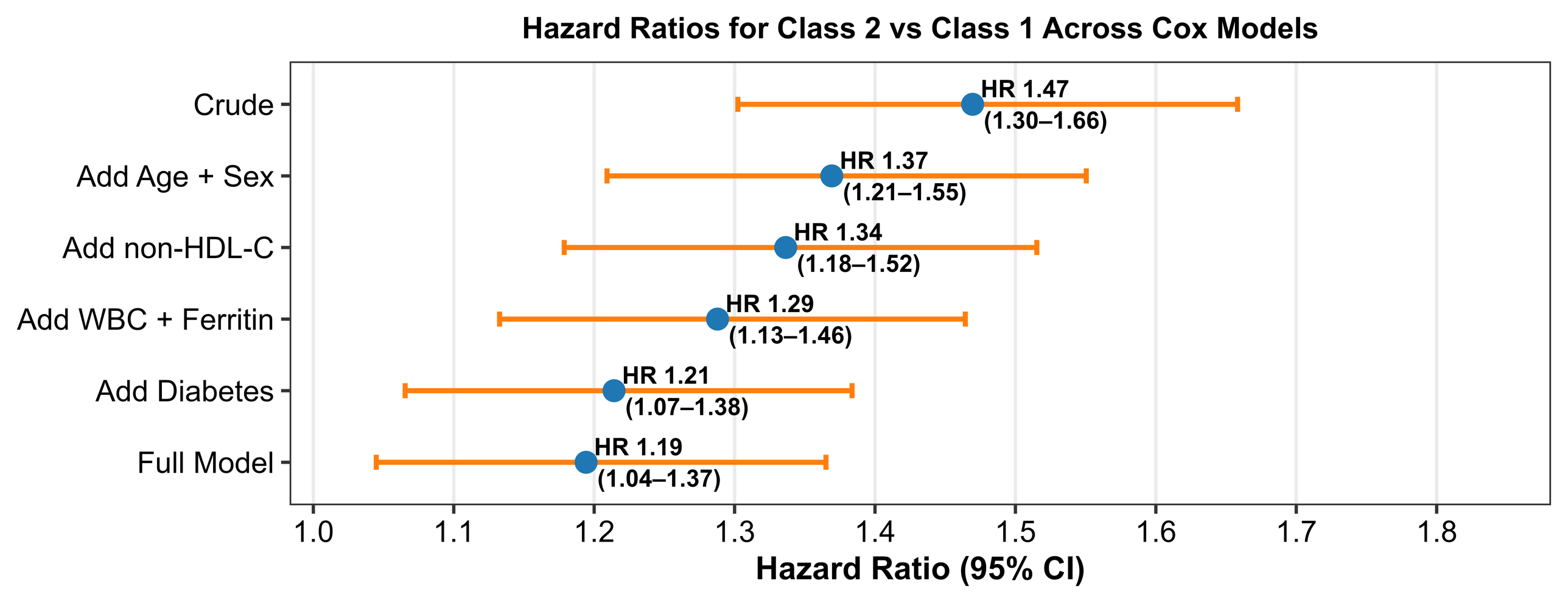

Supplement: Supplementary Figure S3 — Forest plot of hazard ratios (HRs) for CAS progression in the rising TyG-BMI trajectory group compared with the stable trajectory group, restricted to participants with at least 24 months of follow-up. Stepwise adjustments were performed for demographic, metabolic, inflammatory, and comorbidity factors. The results remained consistent with the primary analysis, supporting the robustness of the association. [file Image3.tiff]
