# Supplementary material for: Longitudinal TyG–BMI trajectories predict carotid atherosclerosis progression in a Chinese retrospective cohort
Source: Front Cardiovasc Med. 2025 Nov 28;12:1672514. doi: 10.3389/fcvm.2025.1672514 (PMC12698576; doi:10.3389/fcvm.2025.1672514)
Supplement: Supplementary Table S1 — Reclassification analyses at 60 months (cfNRI and IDI). [file Table1.pdf]

Supplementary Table S1. Reclassification analyses at 60 months (cfNRI and IDI).

Metric	Estimate	95% CI	p-value
**IDI**	0.0023	0.0002–0.0045	0.029
**cfNRI**	0.244	0.151–0.335	<0.001

Category-free Net Reclassification Improvement (cfNRI) and Integrated Discrimination Improvement (IDI) were calculated to evaluate whether adding TyG–BMI trajectory class to the baseline Cox model improved risk classification for CAS progression at 60 months. Both cfNRI and IDI indicated significant improvement in model performance.
